# Critical evaluation of the agro-ecological system of the Republic of South Africa (30°S) in response to unclassified claims of cocoa farming beyond 20°S of the equator

**DOI:** 10.1371/journal.pone.0289873

**Published:** 2023-08-15

**Authors:** Peter Mudiaga Etaware

**Affiliations:** Department of Botany, Faculty of Science, University of Ibadan, Ibadan, Oyo State, Nigeria; University of Ilorin, NIGERIA

## Abstract

Cocoa is a climate sensitive species that has never been reported to grow or survive outside its natural climate belt (20°N-20°S of the equator). Recent reports claimed that cocoa is currently cultivated in Eswatini (26°S), Botswana (22°S), Namibia (22°S), Lesotho (29°S), and the Republic of South Africa “RSA” (30°S). How true are these reports? Climatological and epidemiological investigations were setup to debunk or support these claims. The clime of RSA was investigated since it was the farthest from the cocoa production clime. A review of the climate data of RSA showed 12.4 and 6.1% increase in night-time and day-time temperatures, respectively i.e., from 9.7 and 24.4°C (1901–1930) to 10.9 and 25.9°C (1991–2020), affirming the influence of global warming. The consistent increase in the moving average from 1901–2021 with a fluctuation in the seasonal variation, validates this research. A global connection was established between climate suitability for cocoa production and cocoa disease/pathogen establishment (r = -0.39, P-value = 0.089) at P<0.05. Further analysis showed that the annual temperature (10.8°C≥Temp≥25.8°C), humidity (62%) and sunshine distribution (8.4hours/month) of RSA was suitable for cocoa farming, even though water availability was below the recommended level (rainfall≥463.6mm and rainy_days≤5days/month), farm irrigation systems are currently in use. Other findings showed that KwaZulu-Natal was 100% suitable for cocoa farming, followed by Gauteng, Mpumalanga (86%), Eastern Cape, Limpopo, Northwest (71%), Free State, Northern Cape, and Western Cape (57%). The estimated black pod disease status of KwaZulu-Natal (8.6%) and Eastern Cape (6.6%) affirmed the conduciveness of RSA for cocoa farming.

## 1. Introduction

Cocoa (*Theobroma cacao*) is a climate-sensitive crop that is strictly cultivated between latitude 20°N and 20°S of the equator [1]. Recently, a series of unclassified reports on the internet claimed that some countries outside the confines of the pre-defined climate zone for cocoa production were currently actively involved in cocoa farming e.g., countries like Lesotho, latitude 29.6100°S [2], Eswatini (Swaziland), latitude 26.5225°S [3], Botswana, latitude 22.3285°S [4], Namibia, latitude 22.9576°S [5], and the Republic of South Africa, latitude 30.5595°S [6]. This is an indication that there could be a reform in the natural climate belt for cocoa production, orchestrated by global warming and climate change experienced worldwide. Furthermore, a historical footage recorded in a cocoa plantation in pre-colonial South Africa, by a British media, showed that cocoa was once cultivated in the Republic of South Africa, between 1920 and 1929 [7], affirming and authenticating the claims reported over the internet. These are few documented evidences suggesting that global cocoa cultivation may have expanded beyond its natural climate belt i.e., 20°N and 20°S of the equator [1] down to the fringes of Southern Africa (≥30°S of the equator). What could be the reason(s) for the global reform of the climate belt for cocoa production? Is it possible that well-known facts about the geographical distribution of climate-restricted species like cocoa could be wrong? Or has the recent scientific advancement in genetic engineering conferred ubiquity to cocoa hybrids used as seed plants? Or is the cocoa species dynamics altered by the recent climate change, such that it is no longer threatened by climate restriction? Are theories of ecological distribution of climate-sensitive species becoming outdated? What if the current global climate change was indeed re-organizing global species distribution such that ecological barriers are broken and an unusual facet for new species evolution theory discovered, based on climate re-alignment with the past? These are few questions that should be investigated scientifically.

Thus, this research was setup to find answers to some of the questions raised. An examination of the current climate status of some countries located within the fringes of the Southern part of the African continent (22°S≤Latitude≤35°S) was conducted to unearth facts and scientific explanations that may support or disprove the claims about the recent conduciveness of the climate of Southern Africa for cocoa production. More so, a pre-evaluation of the possibility or likelihood of black pod disease outbreak within that zone was conducted to validate these claims as cocoa pests and pathogens can only thrive or survive in climate zones conducive for cocoa production [8], therefore, a 0% chance of potential disease outbreak will totally disprove the claim of Southern Africa being conducive for cocoa farming. Furthermore, the disease analysis would shed more light on the sustainability of cocoa business within that zone i.e., the pre-evaluated level of disease outbreak and potential crop loss would serve as an avenue for farmers and investors to be able to predict their profit or loss (financially) before the beginning of each cocoa production season. Also, the disease pre-assessment study will provide farmers and investors with a quantitative idea of the cost of cocoa production before funds are even allocated for the project i.e., a succinct pre-budget plan for the management of diseases and pests affecting the cocoa plant can be structured from the information generated and calculated alongside other financial costs for cocoa production. Finally, the climate suitability study and disease pre-assessment test carried out would serve as a tool to pre-quantify the cocoa production capacity of Southern Africa, amidst pestilence. Presently, none of the countries located within Southern Africa like Angola, Namibia, the Republic of South Africa, Mozambique, Swaziland, Zambia, Botswana, Zimbabwe or Lesotho was listed among the top ten (10) producers of cocoa in Africa [9, 10], neither were they among the top thirty (30) cocoa producing nations of the world [10], with the exception of Madagascar, ranked 8^th^ in Africa and 20^th^ in the world (Table 1). Maybe, the outcome of this research would expose the hidden potentials for massive cocoa production in Southern Africa, which would be convincing enough to lure farmers and other foreign investors into cocoa farming, not just in West Africa alone but also in the fertile lands of Southern Africa. The aim of this study was to boost cocoa production in Africa by exploring new ecological zones that could be used for cocoa farming through climate analysis and black pod disease (BPD) of cocoa pre-evaluation, since BPD is the most lethal disease affecting cocoa in Africa, limiting crop yield to almost 0%, in severe situations {10]. It is noteworthy to mention that black pod disease of cocoa thrives better in an environment where the temperature distribution lies between 10–35°C i.e., 10°C≤Minimum_Temperature≤18°C, 18°C≤Average_Temperature≤27°C, and 30°C≤Maximum_Temperature≤35°C; the amount of rainfall exceeds 50 mm per month, while the relative humidity is more than 50%, with an even distribution of sunshine greater than 3 hours per day, and the length of rainfall duration exceeds 6 days per month [10]. Therefore, if this research was able to prove, beyond any doubt, that the agro-ecological zone of the Southern part of Africa was indeed suitable and conducive for cocoa production, then there will be a sizeable increase in the agro-ecological landmass for cocoa production in Africa, which will in turn increase the continent’s cocoa production capacity and also reduce the demand pressure on the global market by increasing its supply capacity.

**Table 1:** An excerpt of some cocoa producing nations of Africa from the global cocoa production statistics.

Global ranking	African ranking	Country	Global cocoa production (tons)	Export %
1^st^	1^st^	Côte d’Ivoire	2,034,000	39.12
2^nd^	2^nd^	Ghana	883,652	16.99
4^th^	3^rd^	Nigeria	328,263	6.31
5^th^	4^th^	Cameroon	295,028	5.67
12^th^	5^th^	Uganda	31,312	0.60
15^th^	6^th^	Togo	22,522	0.43
17^th^	7^th^	Sierra Leone	14,670	0.28
20^th^	8^th^	Madagascar	11,010	0.21
21^st^	9^th^	Guinea	10,638	0.20
22^nd^	10^th^	Liberia	8,552	0.16
23^rd^	11^th^	Tanzania	8,548	0.16
28^th^	12^th^	Republic of Congo	4,000	0.08
29^th^	13^th^	DR Congo	3,758	0.07
30^th^	14^th^	Sao Tome and Principe	2,778	0.05
39^th^	15^th^	Angola	442	0.01
41^st^	16^th^	Equatorial Guinea	413	0.01

**Source**: Etaware [10]

## 2. Methodology

### 2.1 Case study for this research

**Fig 1:**
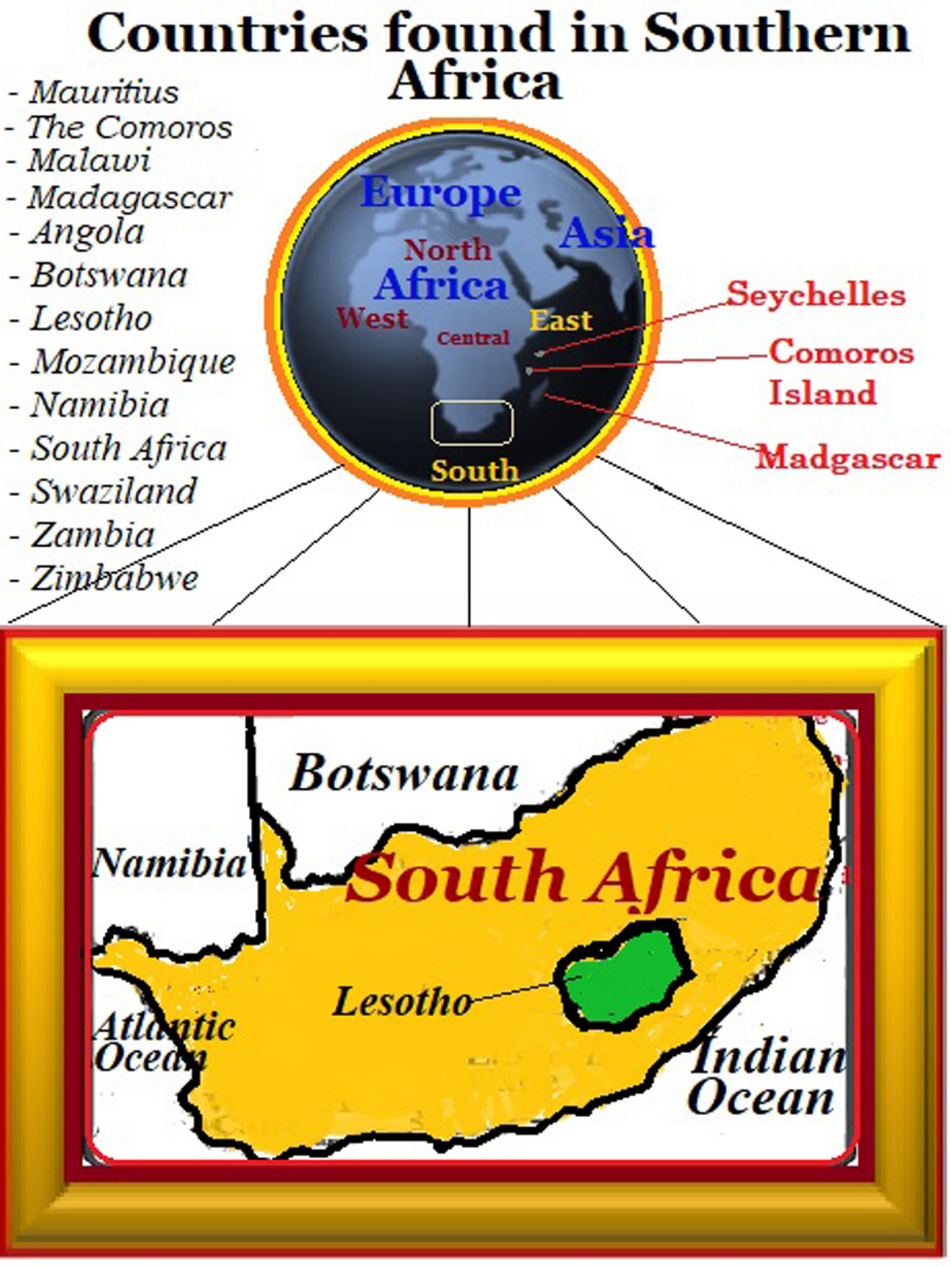
The Republic of South Africa (case study) and some of its neighboring countries.

The Republic of South Africa (RSA) is geographically positioned on the fringes of the African continent i.e., Latitude: 30.5595°S and Longitude: 22.9375°E (Fig 1). The country is bounded to the south by the South Atlantic and Indian Oceans, to the north by Botswana and Zimbabwe, to the east by Mozambique and Swaziland, to the west by Namibia, and at the middle by Lesotho, a centrally circumscribed country [11, 12]. It’s borders spread over 121,909,000 ha, with land area of 121,309,000 ha, of which 96,341,000 ha is used for agriculture, while 17,086,490 ha is covered by forest vegetation and the remainder is either used for housing and other construction works, or encroached by desert [13].

### 2.2 Clime suitability test for cocoa farming using climate data

A comparative assessment was carried out to establish the possibility of the Republic of South Africa to match the cocoa production output capacity (ton-for-ton) of the best cocoa producing nation in the world (Côte d’Ivoire) using climatological data. Also, both nations were subjected to climate suitability studies for cocoa production using the minimum global climate standard for cocoa production. Climate data source for the Republic of South Africa was majorly obtained from the database of Climate Change Knowledge Portal [14], and WorldData [15], while that of Côte d’Ivoire was collected from the database of WorldData [16], and Climate Change Knowledge Portal [17], and that of KwaZulu-Natal Province was obtained from Weather and Climate [18] and WorldData [19], respectively. The secondary aim of the climatological investigation carried out was to compare the clime of the major provinces of the Republic of South Africa with the minimum clime requirement for cocoa production in order to establish and select potential productive land sites, within the Republic of South Africa, that can support maximum cocoa production, with the primary focus on improving the continent’s supply quota or contribution to the global market. The formula provided below was used to determine the climate suitability for cocoa production (CSCP) in this research.


$$\text{CSCP}\left(\%\right)=\frac{\text{The}\;\text{number}\;\text{of}\;\text{cilmate}\;\text{factors}\;\text{that}\;\text{correspond}\;\text{with}\;\text{the}\;\text{standard}\;\text{values}}{\text{The}\;\text{total}\;\text{number}\;\text{of}\;\text{standard}\;\text{cilmate}\;\text{values}\;\text{provided}\;\text{for}\;\text{this}\;\text{assessment}}*100$$

Therefore,

$$\text{CSCP}\left(\%\right)=\frac{n}{7}*100$$

**Note**: Seven (7) standard climate factors for cocoa production were used for this assessment.

### 2.3 Epidemiological survey as confirmatory tool for climate suitability for cocoa production

Black pod disease (BPD) of cocoa is the major biological limitation to bountiful cocoa production in Africa [10, 20–22]. Therefore, the BPD status of the earmarked zones was used as an indicator to pre-evaluate the chances of survival of the pathogen(s) and the level of pestilence on cocoa that should be expected within Southern Africa. The disease appraisal system used for BPD valuation was ETAPOD, a virtual system for measuring black pod disease outbreak, developed by Etaware [20] and upgraded by Etaware *et al*. [22].

### 2.4 Data analysis

Climate maps were drawn using the 2-dimensional contour model (2-DCM) for climate layering and annotations on the cartographic designs were made by Paint App. The cartographic tool for map design was from Minitab 16.0 Statistical software, while the disease appraisal system used for the epidemiological analysis i.e., ETAPOD, a virtual system for black pod disease appraisal and forecast, was developed by Etaware [20] and validated by Etaware *et al*. [22]. The comparison between the dataset was done by defining the percentage increase in climate variables along a stipulated period, 3-point moving averages (MA) and seasonal variation (SV) were also calculated using excel worksheet 2010. The Pearson’s Product Moment of Correlation coefficient (PPMCC) was used to establish the relationship between the climatological survey and the disease epidemiological analysis carried out in this research at P<0.05. Pictorial illustrations (2D-Pictures) were developed using Paint App too.

## 3. Results

### 3.1 The effects of global warming on the cold climates of Southern Africa

The Republic of South Africa (RSA), located at the borderline of the African continent, was the most suitable country for this assessment. The results obtained show that there was a steady increase in the mean annual temperature (MAT) of RSA within a 30-year interval or timeframe i.e., 2.9% increase in MAT from 1931 to 1960, 0.6% increase in MAT between 1961 and 1990, and 4.0% increase in MAT from 1991 to 2020 (Table 2). Also, the same pattern of increase was recorded for the night time (minimum temperature “Min.T”) and day time (maximum temperature “Max.T”) temperatures of the country, within the same timeframe i.e., 3.1% increase in Min.T from 1931 to 1960, 3.0% increase in Min.T from 1961 to 1990, and 5.8% increase in Min.T from 1991 to 2020 (for night time temperature), 2.5% increase in Max.T from 1931 to 1960, no increase in Max.T between 1961 and 1990, and 3.6% increase in Max.T from 1991 to 2020 (for day time temperature), as shown in Table 2.

**Table 2:** Temperature variation along the years within the clime of the Republic of South Africa.

Year	Dec.-Feb.	March-May	June-August	Sept.-Nov.	Annual Mean	% Increase
Mean Annual Temperature °C						
1991–2020	23.59	18.41	12.37	18.98	18.3	4.0
1961–1990	22.93	17.65	11.71	18.29	17.6	0.6
1931–1960	22.73	17.54	11.62	18.14	17.5	2.9
1901–1930	22.41	16.99	11.03	17.6	17.0	0.0
Minimum Temperature °C						
1991–2020	16.7	11.13	4.31	11.26	10.9	5.8
1961–1990	16.1	10.63	3.82	10.84	10.3	3.0
1931–1960	15.73	10.38	3.54	10.46	10.0	3.1
1901–1930	15.55	9.9	3.08	10.07	9.7	0.0
Maximum Temperature °C						
1991–2020	30.53	25.73	20.46	26.74	25.9	3.6
1961–1990	29.81	24.73	19.63	25.8	25.0	0.0
1931–1960	29.77	24.75	19.75	25.87	25.0	2.5
1901–1930	29.33	24.13	19.02	25.17	24.4	0.0

A 10-year analysis of the mean annual temperature of the Republic of South Africa showed that there was a steady decline in the mean annual temperature of the environment between 1901 and 1921 (i.e., 0.66% decline in the ambient temperature). In 1931, the temperature rose by 5.11% and 1.43% in the following year. A dramatic increase and decrease in the mean temperature was observed between 1951 and 1981, and finally, there was a steady increase from 1991 to 2021, with its peak value recorded in 1991 (3.86%), as shown in Table 3. The 3-point moving average estimated for the mean annual temperature of RSA showed consistency in temperature increase from 1901 to 2021, while there was a bit of fluctuation in the mean annual temperature values per season, as described by the level of seasonal variations estimated in Table 3. The highest temperature variation per season was recorded in 1951 (ΔTemperature = 0.34°C), while the least variation was observed in 2001 (ΔTemperature = 0.01°C), as shown in Table 3.

**Table 3:** Temperature variation along the years within the clime of the Republic of South Africa.

Year	Mean Annual Temperature	% Increase	Moving Average (3 Point)	Seasonal Variation
1901	16.87	0.00		
1911	16.76	-0.65	16.76	0.00
1921	16.65	-0.66	16.97	-0.32
1931	17.50	5.11	17.30	0.20
1941	17.75	1.43	17.46	0.29
1951	17.12	-3.55	17.46	-0.34
1961	17.50	2.22	17.29	0.21
1971	17.24	-1.49	17.28	-0.04
1981	17.10	-0.81	17.37	-0.27
1991	17.76	3.86	17.56	0.20
2001	17.81	0.28	17.82	-0.01
2011	17.89	0.45	17.92	-0.03
2021	18.06	0.95		

### 3.2 Is the current climate of Southern Africa conducive for cocoa farming?

Is it possible for the cocoa tree to survive and produce quality cocoa beans outside its natural climate belt i.e., Latitude 20°N and 20°S of the equator? How possible is it for the clime of the Republic of South Africa, located in the fringes of the Southern part of the African continent beyond 30°S of the equator, to naturally sustain cocoa farming? The investigation carried out during the dry months of the year showed that the clime of the Republic of South Africa (RSA) was 100% suitable for cocoa cultivation between January and March, as 7 out of its 7 investigated climatic factors, stated in Table 4, were within the range of the standard global clime or above the lowest climate requirement for cocoa production, with 100% climate suitability ratio for cocoa farming (Table 4). In contrast, the clime of the best cocoa producing nation of the world i.e., Côte d’Ivoire, was not totally conducive for cocoa production between January and March, with 71, 57 and 71% climate suitability ratio for January, February and March, respectively (Table 4). There was a shift in climate and terrain suitability for cocoa farming between Côte d’Ivoire and RSA as the investigation conducted during the rainy season in Africa, showed that in April and May, the clime of Côte d’Ivoire was more suitable for cocoa production (86% climate estimation) than that of RSA with 71 and 43% climate suitability ratio for cocoa farming, respectively (Table 4). The poor clime for cocoa farm activities in RSA still continued from June down to October, with 43% climate suitability ratio estimated for cocoa production between June—September, and an upgrade to 71% in October. That of Côte d’Ivoire was 100% suitable for cocoa cultivation between the months of June and October, as 7 out of its 7 investigated climatic factors were within the range of the standard global clime or above the lowest climate condition for cocoa production, with a potential climate rating of 100% for cocoa production (Table 4). For the periods within the onset of the dry season in Africa i.e., November and December, it was noticed that the climate and terrain of RSA was more suitable and adaptable for cocoa production than that of Côte d’Ivoire, as none of the climate factors inspected were below the standard or lowest requirements for cocoa farming i.e., 7 perfect climate match out of the 7 outlined climatic factors pertinent for optimum cocoa production i.e., 100% suitability score was recorded for the months of November and December for RSA, while 100 and 71% climate suitability ratio for Cote d’Ivoire within the same period, as shown in Table 4.

**Table 4:** Climate comparison between the world best cocoa producing nation (Côte d’Ivoire) and the Republic of South Africa.

**Duration**	**Minimum Temperature (** ^**o**^ **C)**		**Average Temperature (** ^**o**^ **C)**		**Maximum Temperature (** ^**o**^ **C)**		**Sunshine duration (Hours)**	
Month	South Africa	Côte d’Ivoire	South Africa	Côte d’Ivoire	South Africa	Côte d’Ivoire	South Africa	Côte d’Ivoire
January	16.7	20.0	23.7	26.3	30.7	34.0	8.4	7.3
February	16.7	22.0	23.5	28.3*	30.3	35.0	8.6	7.4
March	14.8	23.0	21.8	29.1*	28.8	35.0	8.1	7.1
April	11.3	23.0	18.4	28.6*	25.6	34.0	7.0	7.2
May	7.3*	23.0	15.0*	27.6*	22.8	33.0	8.3	6.8
June	4.1*	22.5	11.9*	26.4	19.8	31.0	8.1	5.1
July	3.6*	22.5	11.6*	25.4	19.8	29.0	8.6	4.1
August	5.3*	22.0	13.5*	25.1	21.8	29.0	8.7	3.4
September	8.5*	22.0	16.6*	25.7	24.8	31.0	8.8	4.6
October	11.6	22.5	19.3	26.5	27.0	32.0	8.8	6.5
November	13.6	22.0	21.0	26.9	28.4	33.0	8.8	7.2
December	15.5	21.0	22.7	26.2	29.9	33.5	8.5	7.2
Mean	10.8	22.1	18.3	26.8	25.8	32.5	8.4	6.2
*Standard clime*	18°C (Lowest = 10°C)		≤27°C (Lowest = 18°C)		30°C (Highest = 35°C)		Lowest = 3 hours	
***Source***	[23, 24]		[25, 26]		[23, 27]		[28]	
**Duration**	**Precipitation/Rainfall (mm)**		**Relative humidity (%)**		**Rainy days/Month**		**Climate match (7 factors)**	
Month	South Africa	Côte d’Ivoire	South Africa	Côte d’Ivoire	South Africa	Côte d’Ivoire	South Africa	Côte d’Ivoire
January	70.9	13.0*	63	66	7.9	1.8*	7/7 (100%)	5/7 (71%)
February	64.8	33.8*	65	66	6.6	3.2*	7/7 (100%)	4/7 (57%)
March	56.9	78.0	68	70	6.2	5.8*	7/7 (100%)	5/7 (71%)
April	32.7*	122.9	68	75	4.5*	8.2	5/7 (71%)	6/7 (86%)
May	18.7*	149.5	64	80	2.6*	10.5	3/7 (43%)	6/7 (86%)
June	13.5*	180.6	63	82	2.3*	11.9	3/7 (43%)	7/7 (100%)
July	12.0*	147.9	60	84	1.9*	9.4	3/7 (43%)	7/7 (100%)
August	13.8*	155,2	58	86	2.3*	11.2	3/7 (43%)	7/7 (100%)
September	18.6*	186.5	57	84	2.6*	12.5	3/7 (43%)	7/7 (100%)
October	38.5*	152.9	59	82	5.4*	12.8	5/7 (71%)	7/7 (100%)
November	55.5	58.2	60	79	7.0	7.9	7/7 (100%)	7/7 (100%)
December	67.7	19.7*	60	72	7.9	2.2*	7/7 (100%)	5/7 (71%)
Mean	39(464)	108(1,298)	62	77	4.8	8.1		
*Standard clime*	100 mm (Lowest = 50 mm)		70% (Lowest = 50%)		Lowest = 6 days		5 out of 12	6 out of 12
***Source***	[10, 23]		[10, 23]		[10]			

Mean climate values with

* superscript are lower than the benchmark values i.e., Standard value and lowest limits (in parenthesis), or higher than the highest possible climate requirement for global cocoa production. Therefore, they are unfavorable for cocoa farming.

**Fig 2:**
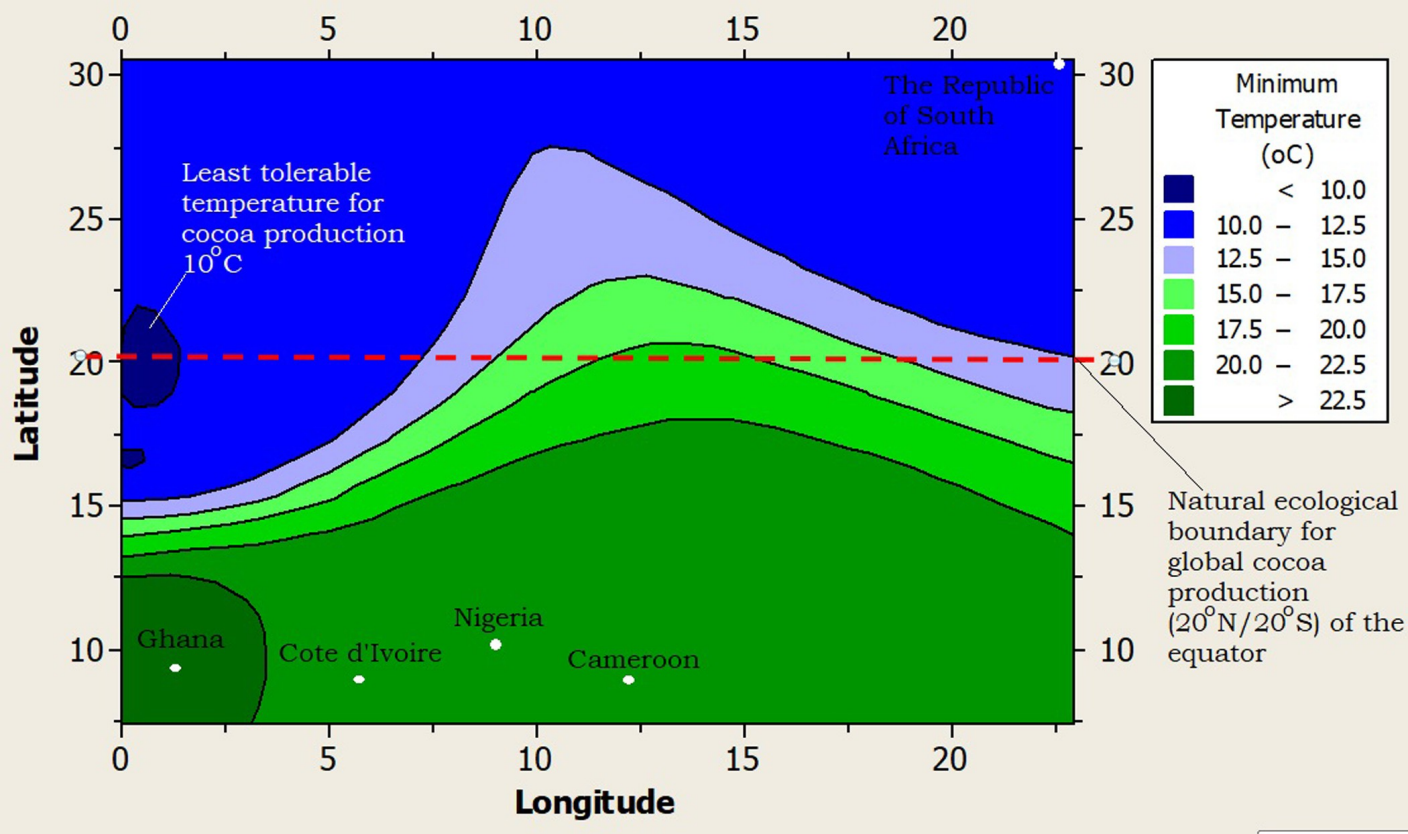
A 2-dimension contour model (2-DCM) for night time temperatures.

**Fig 3:**
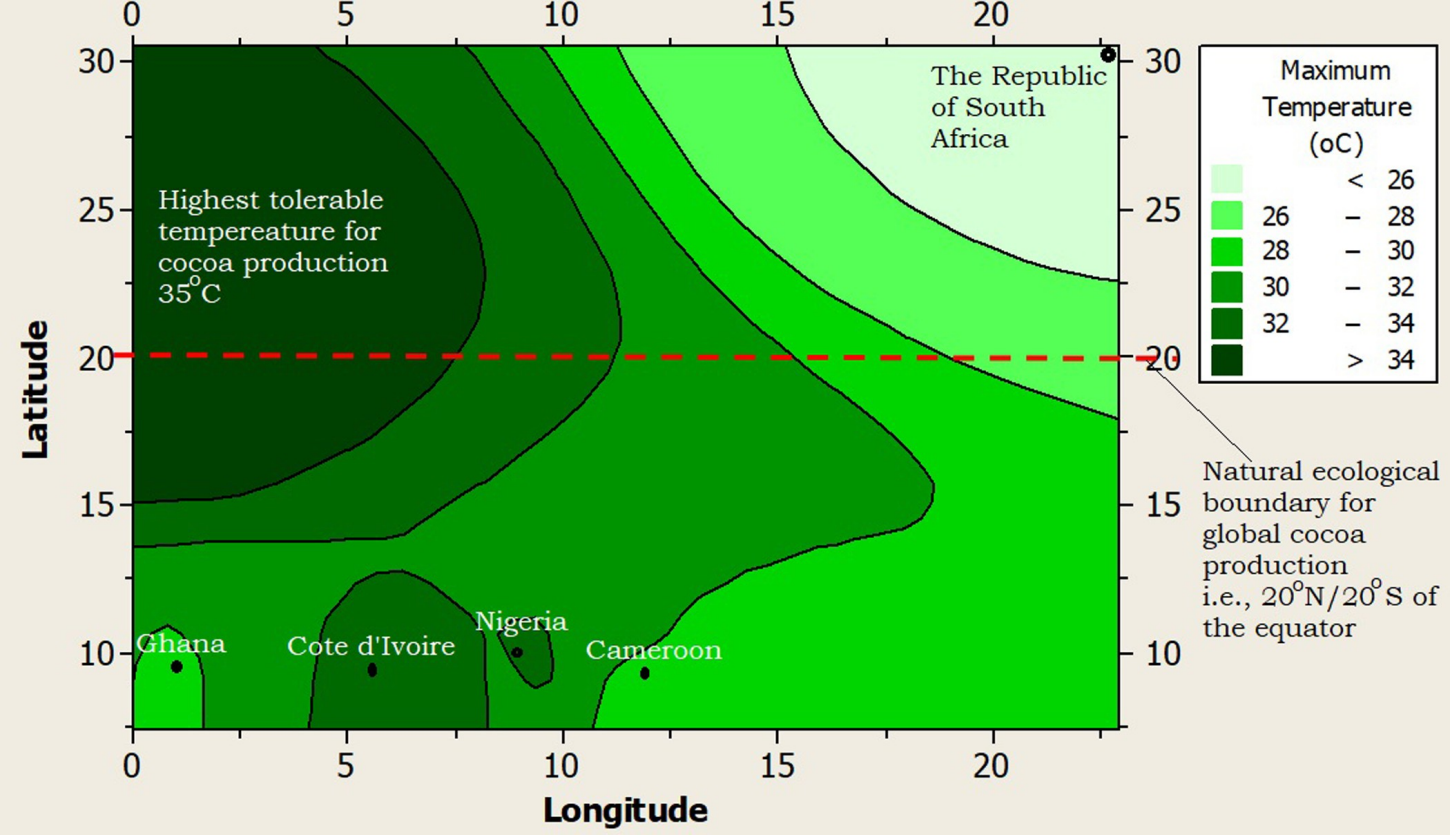
A 2-dimension contour model (2-DCM) for day time temperatures.

**Fig 4:**
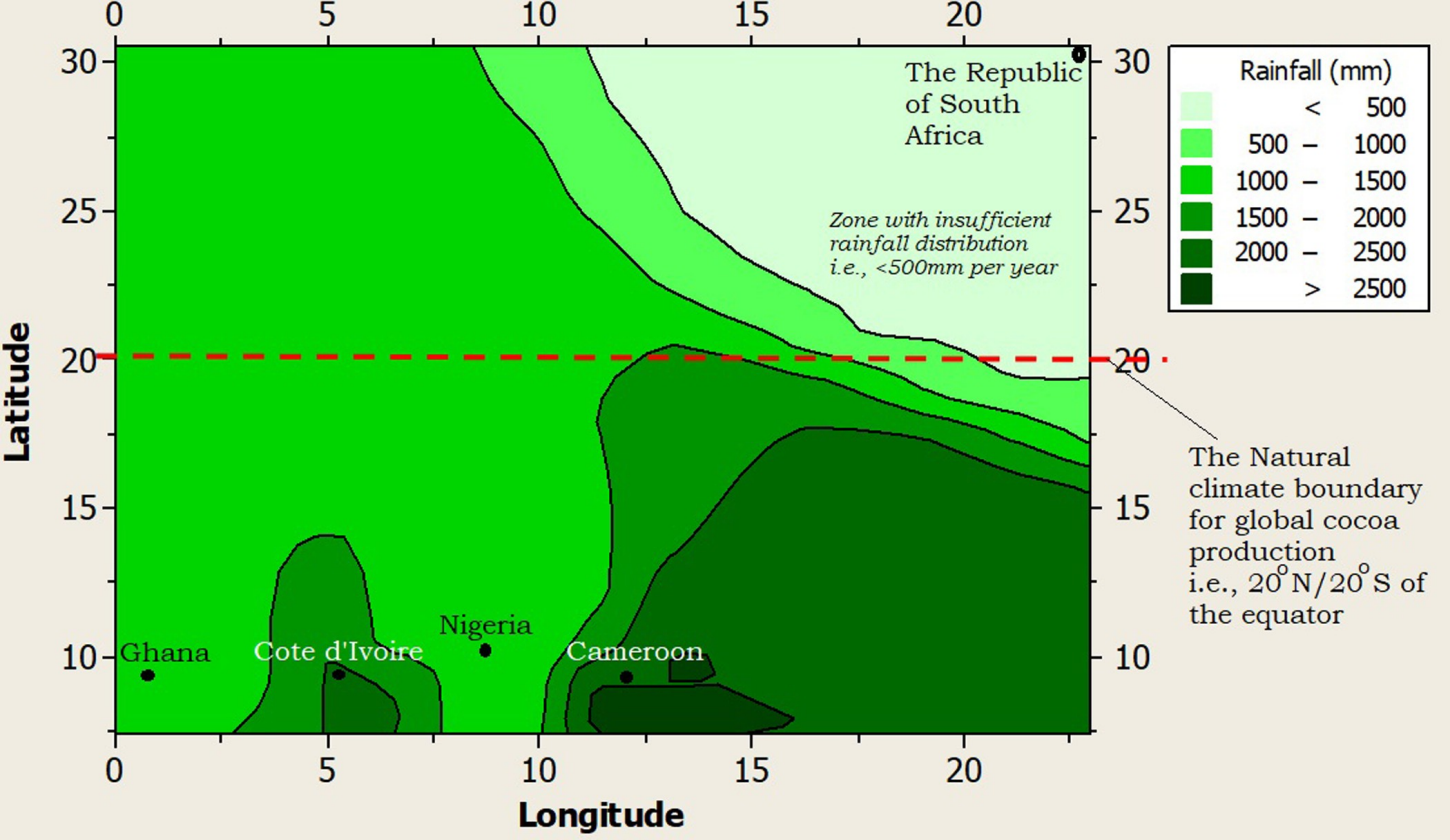
A 2-dimension contour model (2-DCM) for rainfall distribution.

**Fig 5:**
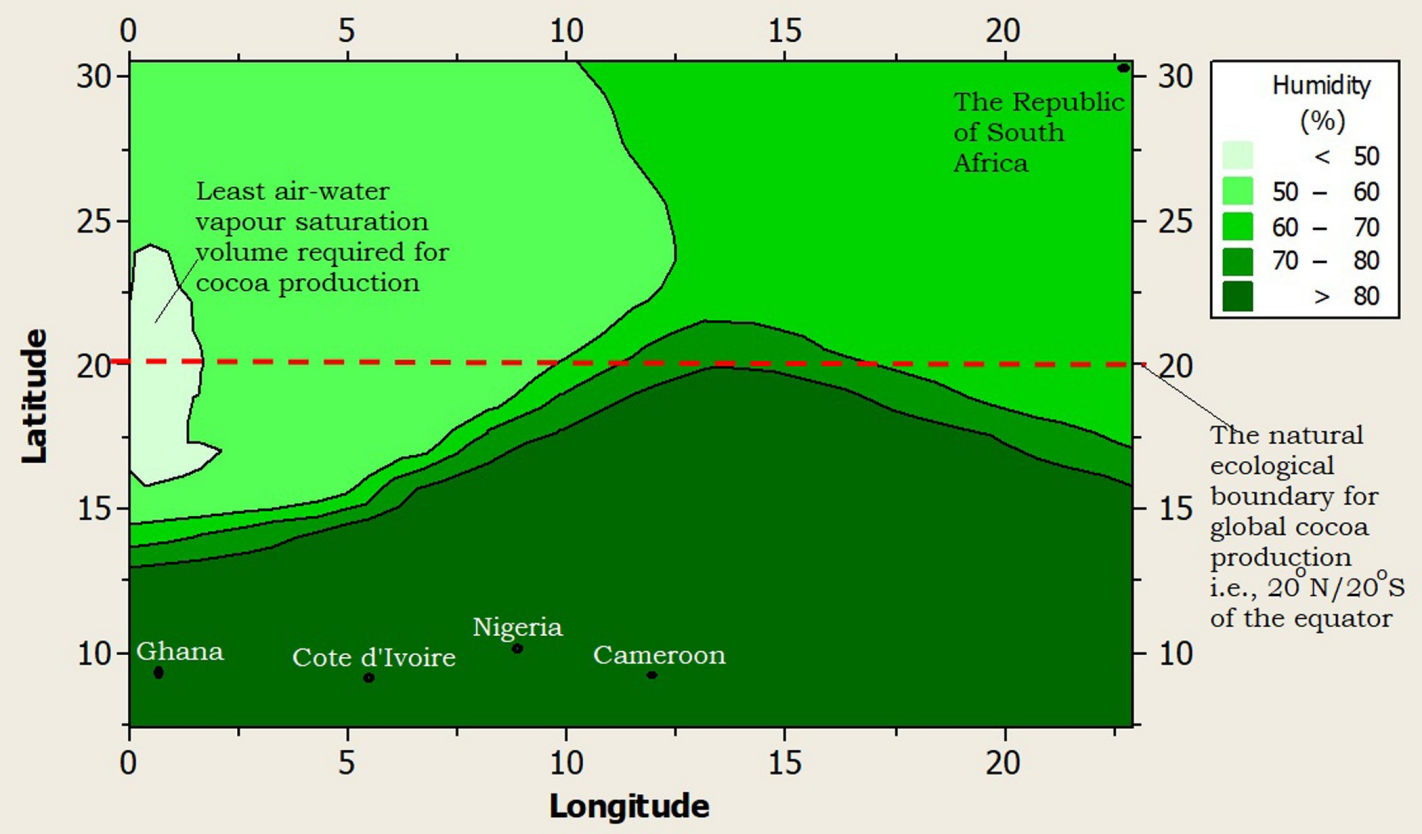
A 2-dimension contour model (2-DCM) for humidity.

Furthermore, the climate of RSA was profiled alongside the top four (4) cocoa producing nations of Africa and the world at large (i.e., Côte d’Ivoire, Ghana, Nigeria and Cameroon), using a 2-dimensional contour model (2-DCM) for climate layering. A juxtaposition of the profiled climates showed that Ghana had the warmest night time temperature (>22.5°C), while Côte d’Ivoire, Nigeria and Cameroon shared similar midnight temperatures (20–22.5°C) as shown in Fig 2. The midnight temperature of RSA was the coldest (10.8°C) but it was still higher than the least tolerable midnight temperature (minimum temperature = 10°C) that the cocoa tree can withstand (Fig 2). The day time or maximum temperature limit for RSA was the best (25.8°C) among all the profiled climates, as it was far below the highest tolerable day time temperature limit for cocoa production (maximum temperature limit = 35°C), and well within the optimum temperature (27°C) favorable for active cocoa farm activities (Fig 3). The rainfall supply in RSA was very poor as it was well below 500mm per year (on the average) compared to the least required rainfall distribution for cocoa production i.e., 1,200mm per year (Fig 4). On the average, the rainfall distribution for the other cocoa producing nations like Ghana (>1,000 mm per year), Côte d’Ivoire (>1,500 mm per year), Nigeria (>1,000 mm per year) and Cameroon (>1,500 mm per year) were above or within the limits of the water requirements for cocoa farming i.e., 1,200 mm per year, as shown in Fig 4. All the profiled climates had substantial amount of air-water vapour saturation volume but that of RSA was the least, with an estimated value 10–20% higher than the least possible humidity requirement for cocoa production (Fig 5) i.e., the humidity of RSA was between 60–70% while the other countries whose climates were profiled alongside were between 70–80% compared to the benchmark (50%), as shown in Fig 5. In summary, the climate of RSA was described thus: temperature range was 10.8°C≥Temp.≥25.8°C, rainfall<500mm, rainy_days<5 days/month, humidity≥62%, and there was abundant distribution of sunshine all year round i.e., 8 hours 24 minutes on the average.

### 3.3 Where can cocoa be cultivated within the Republic of South Africa?

Almost all the agricultural areas in the Republic of South Africa was adjudged (by climate estimation) to be able to sustain commercial cocoa cultivation to some extent, but the Province with the best clime for maximum cocoa production was KwaZulu-Natal with a perfect score of 7 out of 7 and a climate suitability ratio of 100% (Table 5). The provinces of Gauteng, and Mpumalanga were the 2^nd^ best for cocoa farming within the country, with 6 out of the 7 climatic factors perfectly aligned with the benchmark for cocoa production i.e., each of the provinces had 86% climate suitability ratio for cocoa farming (Table 5). Eastern Cape, Limpopo, and Northwest provinces were the 3^rd^ best areas for cocoa farming in the Republic of South Africa, with 71% climate suitability ratio for cocoa production i.e., each of the provinces had 5 suitable and 2 unsuitable climatic factors compared to benchmark criteria (Table 5). The least considered landmarks for cocoa farm establishment within the Republic of South Africa were the Free State, Northern Cape, and Western Cape Provinces with only 57% climate suitability ratio for cocoa production. Sadly, only 4 of their climatic factors were perfectly aligned with the benchmark criteria for cocoa production, as shown in Table 5.

**Table 5:** Climate suitability studies for cocoa farming within the nine (9) Provinces of the Republic of South Africa.

		Average Annual Weather/Climate Data for the Republic of South Africa								
		Temperature (°C)			Rainfall	Humidity	Sunshine	Rainy days	Climate match	CSCP (%)
^S^/_N_	Province	Maximum	Average	Minimum	(mm)	(%)	(Hours)		(7 Factors)	
1	Eastern Cape	23.9	18.2	12.5	48.3*	77	8.3	5.3*	5/7	71
2	Gauteng	24.4	18.2	11.9	55.3	56	8.5	4.6*	6/7	86
3	KwaZulu-Natal	25.3	21.0	16.7	76.3	77	6.7	7.1	7/7	100
4	Limpopo	25.9	19.0	12.1	36.8*	57	8.5	3.3*	5/7	71
5	Mpumalanga	25.1	18.9	12.6	59.0	57	8.6	4.6*	6/7	86
6	North-West	24.7	17.7*	10.7	52.6	56	8.8	4.7*	5/7	71
7	Northern Cape	26.4	18.5	10.6	22.5*	48*	9.9	2.7*	4/7	57
8	Free State	24.1	15.7*	7.2*	51.1	56	8.7	5.2*	4/7	57
9	Western Cape	23.2	17.0*	10.7	35.9*	66	8.6	4.7*	4/7	57
	*Standard clime*	30°C	≤27°C	18°C	100 mm	70%	≥3 hours	≥6 days	7/7	100
	***Source***	[23]	[25]	[23]	[23]	[23]				
	*Lowest/Highest*	35°C	18°C	10°C	50 mm	50%	3 hours	6 days		
	***Source***	[27]	[26]	[24]	[10]	[10]	[28]	[10]		

Mean climate values with

* superscript are lower than the benchmark values i.e., Standard value and lowest limits (in parenthesis), or higher than the highest possible climate requirement for global cocoa production. Therefore, they are unfavorable for cocoa farming.

**Note**: CSCP (%)–The percentage of the climate suitable for cocoa production

### 3.4 How suitable is the clime of KwaZulu-Natal Province for cocoa farming?

The climate of KwaZulu-Natal province was compared with the standard climatic factors for global cocoa production in order to determine if it can truly sustain maximum cocoa production. It was observed that the best months for cocoa production within the KwaZulu-Natal province were January, February, March, April, May, July, September, November and December with 100% climate suitability rating for cocoa production i.e., a perfect climate score of 7 out of 7 was reported for those months (Table 6). The months of June, August, and October were also adjudged to be conducive for cocoa farming too (86% climate suitability ratio for cocoa production), but each of these months had one unsuitable climate factor for cocoa production i.e., There was inadequate supply of moisture (rain water) to the environment to sustain cocoa growth during the months of June and August, as the monthly precipitation or rainfall value was well below the lowest tolerable limit (50 mm) for the cocoa plants i.e., 35.2 and 45.0 mm, respectively. For October, the maximum temperature of the environment (36°C) was a little bit above the highest recommended temperature value (35°C) for commercial cocoa cultivation (Table 6).

**Table 6:** Climate feasibility studies for cocoa farming in KwaZulu Natal Province, Republic of South Africa.

	Temperature (°C)			Rainfall	Rainy days	Humidity	Sunshine	Climate match	CSCP (%)
Month	Maximum	Average	Minimum	(mm)		(%)	(Hours)	(7 Factors)	
January	35.0	25.5	19.0	179.7	19.2	79.2	11.9	7/7	100
February	35.0	25.8	18.0	142.9	15.3	78.9	11.9	7/7	100
March	33.0	25.3	14.0	144.0	16.6	79.3	10.7	7/7	100
April	34.0	23.4	15.0	93.3	13.1	75.7	7.5	7/7	100
May	32.0	22.2	13.0	77.3	9.5	72.8	7.9	7/7	100
June	31.0	20.5	12.0	35.2*	6.9	65.7	8.1	6/7	86
July	29.0	19.9	10.0	61.6	7.9	68.1	7.9	7/7	100
August	33.0	20.7	10.0	45.0*	7.4	68.7	7.9	6/7	86
September	35.0	21.7	10.0	77.3	10.2	73.1	9.1	7/7	100
October	36.0*	22.0	14.0	127.9	16.7	76.0	11.1	6/7	86
November	33.0	23.0	14.0	158.6	18.4	78.5	11.6	7/7	100
December	35.0	24.5	13.0	136.0	18.6	80.1	11.9	7/7	100
*Standard clime*	30°C	≤27°C	18°C	100 mm	≥6 days	70%	≥3 hours	7/7	100
***Source***	[23]	[25]	[23]	[23]		[23]			
*Lowest/Highest*	35°C	18°C	10°C	50 mm	6 days	50%	3 hours		
***Source***	[27]	[26]	[24]	[10]	[10]	[10]	[28]		

Mean climate values with

* superscript are lower than the benchmark values i.e., Standard value and lowest limits (in parenthesis), or higher than the highest possible climate requirement for global cocoa production. Therefore, they are unfavorable for cocoa farming.

**Note**: CSCP (%)–The percentage of the climate suitable for cocoa production

### 3.5 Epidemiological evaluation of the global cocoa situation

How accurate and reliable is the climate suitability analysis carried out on the Republic of South Africa? If truly cocoa can be cultivated in the Republic of South Africa, would it be possible that the country will share similar black pod disease epidemiology with other cocoa producing nations within and outside Africa? Is it even possible for the pathogens of cocoa e.g. *Phytophthora* spp., *Moniliophthora* spp., *Botryodiplodia* spp., and cocoa swollen shoot virus (CSSV), to exist outside an environment that is unfavorable to their host plant(s)? Therefore, the epidemiological analysis was setup to answer the questions raised above and also to validate the results obtained from the climatological studies carried out in the previous sections of this article. The disease epidemiological assessment was conducted in four (4) different phases to establish the relatedness between climate suitability for cocoa farming and the propensity for cocoa disease outbreak. The phases were listed below:

▪ Global
▪ Continental (Africa)
▪ National (The Republic of South Africa)
▪ Provincial

#### 3.5.1 Global black pod disease epidemiology

**Fig 6:**
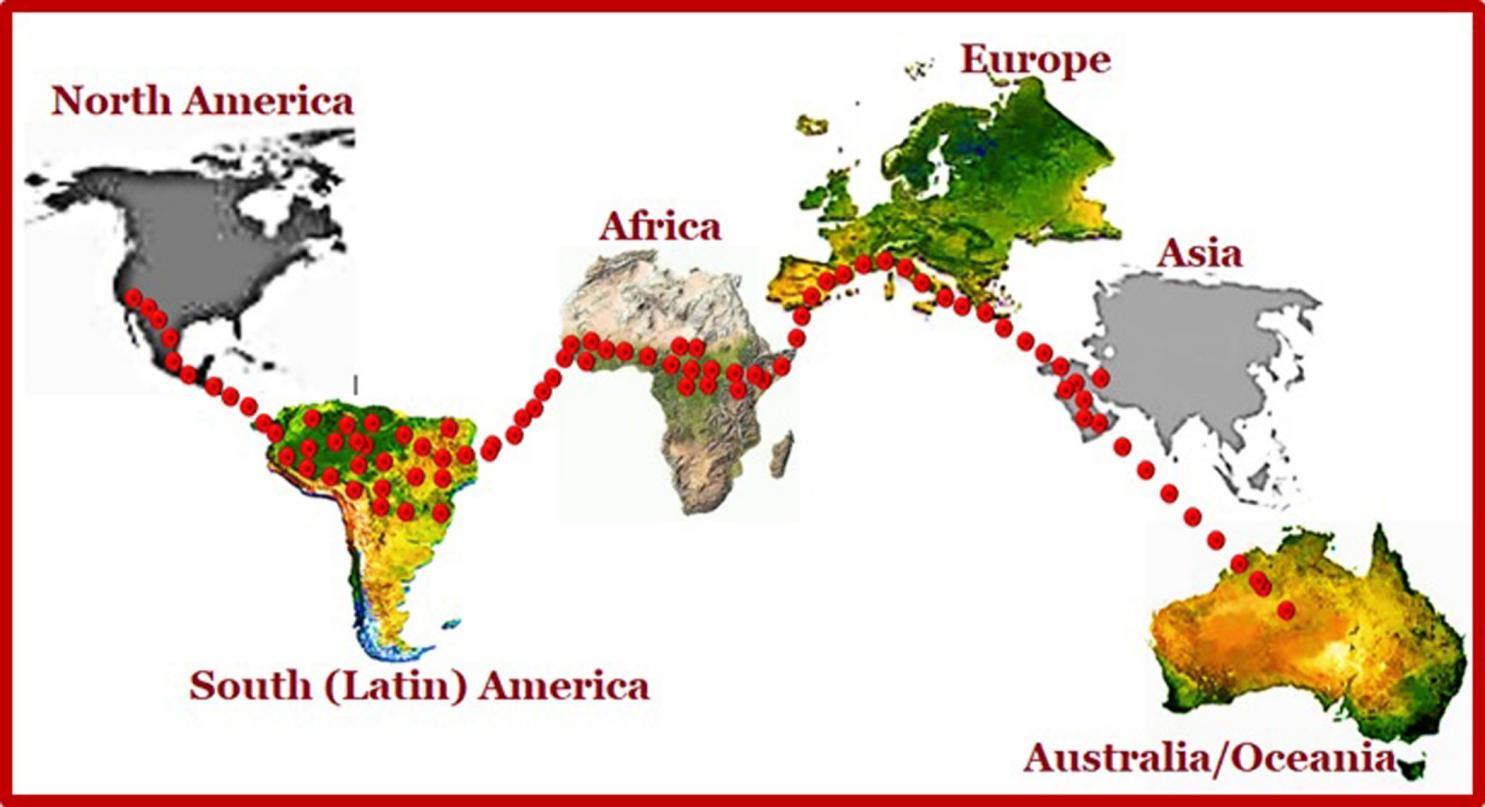
Global trend for black pod disease outbreak.

There was a global connection (r = -0.39, P-value = 0.089) between black pod disease (BPD) status and climate suitability for cocoa farming (using the global ranking for cocoa production as a reference for clime suitability) at P<0.05 (Table 7). It was observed that none of the top 20 cocoa producing nations of the world had 0% prediction for black pod disease (BPD) occurrence (Table 7). The least evaluated situation of BPD outbreak was in Mexico, North American Continent, with 2.1% annual BPD outbreak (Table 7). The highest likelihood of BPD outbreak within the top 20 most productive cocoa nations was recorded in Ghana, with an all-time highest value of 14.6% predicted outbreak of the disease (Table 7). The level of BPD outbreak in the best cocoa producing nation of the world (Côte d’Ivoire), located in Africa, was also adjudged to be relatively high too, with a value of 13.2%, and the least cocoa producing country (Madagascar, located in the Southern part of the African continent), ranked 20^th^ on the list, was also adjudge to have a high value for black pod disease outbreak annually i.e., 11.1% BPD occurrence (Table 7). Other countries like Cameroon (13.4%), Uganda (13.3%), Dominican Republic (13.1%), and Indonesia (13.0%) had higher chances of BPD outbreak (Table 7). A theoretical pattern or trend of the pathogens’ distribution and spread around the world was shown in Fig 6. The pattern created was based on the epidemiological survey and the disease status projected for each continent, as it was indeed possible that the pathogens’ population density would be more in Africa and Latin America, than any other continent in the world, as described in Fig 6.

**Table 7:** The burden of black pod disease in the top 20 cocoa producing nations of the world.

Global ranking	Country	Continent	BPD Status (%)	r	P-Value
1^st^	Côte d’Ivoire	Africa	13.2	-0.39	0.089
2^nd^	Ghana	Africa	14.6		
3^rd^	Indonesia	Asia	13.0		
4^th^	Nigeria	Africa	12.9		
5^th^	Cameroon	Africa	13.4		
6^th^	Brazil	Latin America	11.6		
7^th^	Ecuador	Latin America	11.2		
8^th^	Peru	Latin America	10.2		
9^th^	Dominican Republic	North America	13.1		
10^th^	Colombia	Latin America	12.2		
11^th^	Papua New Guinea	Oceania/Australia	11.4		
12^th^	Uganda	Africa	13.3		
13^th^	Mexico	North America	02.1		
14^th^	Venezuela	Latin America	11.4		
15^th^	Togo	Africa	08.0		
16^th^	India	Asia	11.1		
17^th^	Sierra Leone	Africa	09.9		
18^th^	Haiti	North America	12.0		
19^th^	Guatemala	North America	12.2		
20^th^	Madagascar	Africa	11.1		

**Source**: Excerpts from the global black pod disease (BPD) burden by Etaware [10]. Statistics: Rank vs. Status

#### 3.5.2 Black pod disease epidemiology in Africa

There was an established relationship (r = -0.349, P-value = 0.185) between black pod disease (BPD) status and the African climate suitability for cocoa farming (using the African continental ranking for cocoa production as a reference for clime suitability) at P<0.05 (Table 8). Also, none of the top 16 cocoa producing nations of Africa was adjudged to have 0.0% potentials or propensity for BPD occurrence or 0.0% affiliation with the causal pathogens (Table 8). The highest evaluation of BPD outbreak in Africa was reported in Ghana, with a pre-evaluated value of 14.6% chance of black pod disease occurrence (Table 8). The level of BPD outbreak in the best cocoa producing nation within the African continent i.e., Côte d’Ivoire, was also adjudged to have a propensity for high annual black pod disease outbreak, with a value of 13.2% estimated using ETAPOD. Other countries like Cameroon (13.4%), Uganda (13.3%), Nigeria (12.9%), Equatorial Guinea (12.1%), Republic of Congo (12.1%), Sao Tome and Principe (12.0), Angola (11.5%) and Madagascar (11.1%), had higher chances of BPD outbreak too (Table 8). The current cocoa producing nation in Africa, ranked among the Top 16 cocoa producers of the continent, with the least profile for black pod disease outbreak was Togo, with an annual estimation of 8.0% probability of black pod disease outbreak (Table 8).

**Table 8:** The burden of black pod disease in some cocoa producing countries in Africa.

Global ranking	African ranking	Country	BPD Status (%)	r	P-Value
1^st^	1^st^	Côte d’Ivoire	13.2	-0.349	0.185
2^nd^	2^nd^	Ghana	14.6		
4^th^	3^rd^	Nigeria	12.9		
5^th^	4^th^	Cameroon	13.4		
12^th^	5^th^	Uganda	13.3		
15^th^	6^th^	Togo	08.0		
17^th^	7^th^	Sierra Leone	09.9		
20^th^	8^th^	Madagascar	11.1		
21^st^	9^th^	Guinea	10.9		
22^nd^	10^th^	Liberia	11.9		
23^rd^	11^th^	Tanzania	10.1		
28^th^	12^th^	Republic of Congo	12.1		
29^th^	13^th^	DR Congo	11.0		
30^th^	14^th^	Sao Tome and Principe	12.0		
39^th^	15^th^	Angola	11.5		
41^st^	16^th^	Equatorial Guinea	12.1		

**Source**: Excerpts from the global black pod disease (BPD) burden by Etaware [10]. Statistics: Rank vs. Status

#### 3.5.3 The chances of recording black pod disease outbreak in the Republic of South Africa

**Fig 7:**
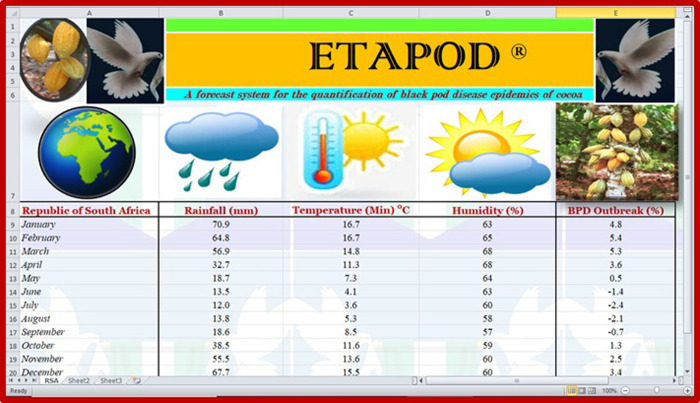
The trend of black pod disease outbreak in the Republic of South Africa.

The epidemiological studies conducted showed that black pod disease would be more intense between January and April every year, if cocoa was actually cultivated in the Republic of South Africa, with a monthly range estimated between 3.6 and 5.4% (Table 9 and Fig 7). The pre-figured level of black disease outbreak (BPD) in the Republic of South Africa (Fig 7) was expected to be at its peak in the month of February (5.4%) and at its lowest in the month of July (-2.4%), as shown in Table 9 below. In any case, the months with low expectation for the disease outbreak were May (0.5%), June (-1.4%), July (-2.4%), August (-2.1%) and September (-0.7%), after which there will be further increase of the disease outbreak between October and December, from 1.3 to 3.4% as shown in Table 9 and Fig 7.

**Table 9:** Black pod disease burden in the Republic of South Africa.

Republic of South Africa	Rainfall (mm)	Temperature (Min)°C	Humidity (%)	BPD Outbreak (%)
*January*	70.9	16.7	63	4.8
*February*	64.8	16.7	65	5.4
*March*	56.9	14.8	68	5.3
*April*	32.7	11.3	68	3.6
*May*	18.7	7.3	64	0.5
*June*	13.5	4.1	63	-1.4
*July*	12.0	3.6	60	-2.4
*August*	13.8	5.3	58	-2.1
*September*	18.6	8.5	57	-0.7
*October*	38.5	11.6	59	1.3
*November*	55.5	13.6	60	2.5
*December*	67.7	15.5	60	3.4

BPD = Black pod disease

#### 3.5.4 The province(s) of the Republic of South Africa that would have been affected by black pod disease outbreak

**Fig 8:**
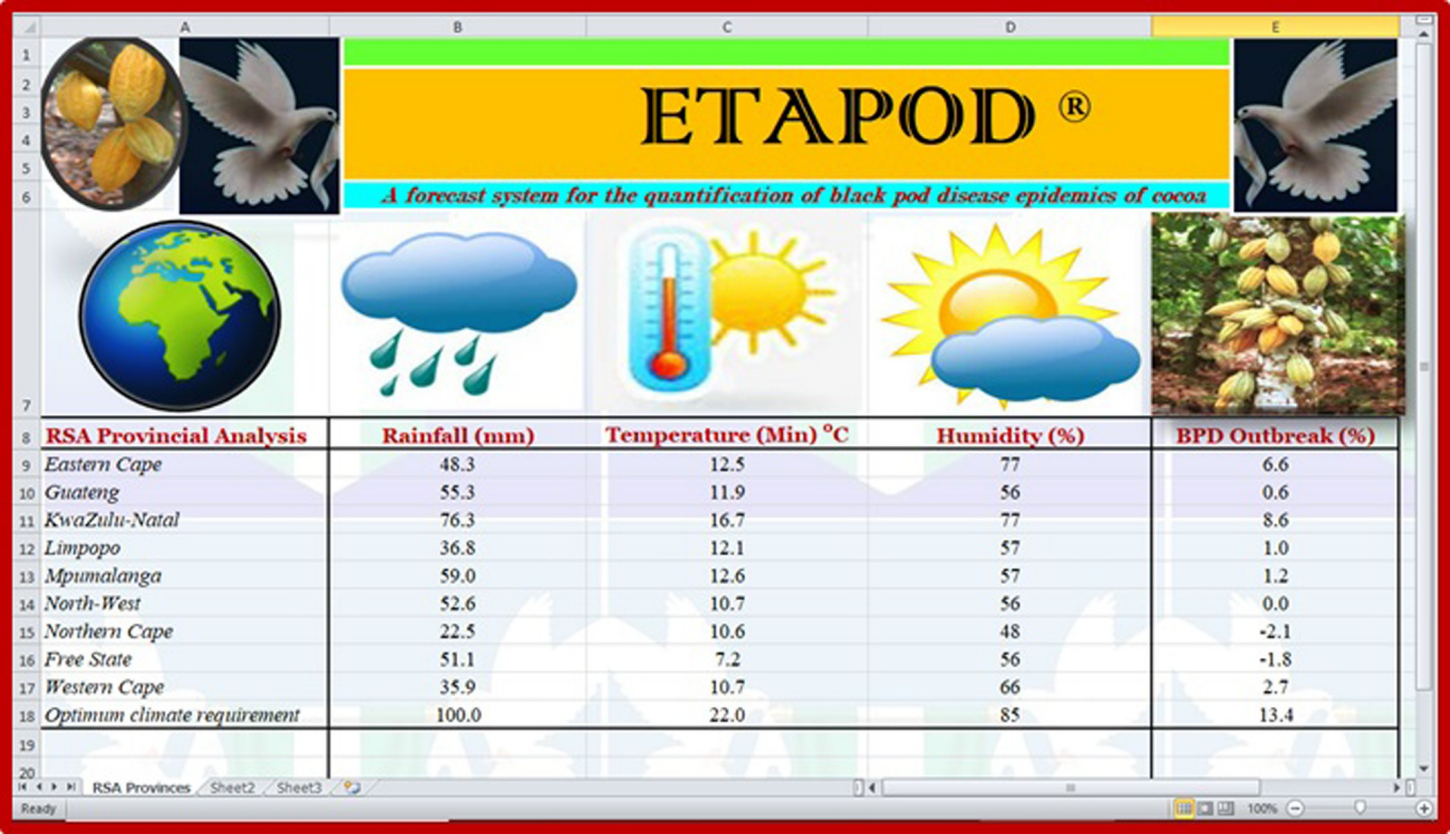
Pre-evaluated levels of black pod disease burden with the major provinces of RSA.

The annual outbreak of black pod disease of cocoa could have been more intense in cocoa plantations situated within KwaZulu-Natal (8.6%) and Eastern Cape (6.6%) provinces of the Republic of South Africa, if cocoa farming was practiced within these provinces (Fig 8 and Table 10). It was also estimated that cocoa farms situated or established in provinces like Guateng, Limpopo, Mpumalanga and Western Cape would have recorded very low annual outbreak of the disease (Fig 8), with annual black pod disease statuses of these areas estimated as 0.6, 1.0, 1.2, and 2.7%, respectively (Table 10 and Fig 8). Finally, there would have not been any issues or concerns regarding the management of black pod disease in areas like North-West (0.0%), Northern Cape (-2.1%) and the Free State (-1.8%), if cocoa farming was actually practiced in these regions (Fig 8 and Table 10).

**Table 10:** Black pod disease burden within the provinces of the Republic of South Africa.

Province	Rainfall (mm)	Temperature (Min)°C	Humidity (%)	BPD Outbreak (%)
*Eastern Cape*	48.3	12.5	77	6.6
*Guateng*	55.3	11.9	56	0.6
*KwaZulu-Natal*	76.3	16.7	77	8.6
*Limpopo*	36.8	12.1	57	1.0
*Mpumalanga*	59.0	12.6	57	1.2
*North-West*	52.6	10.7	56	0.0
*Northern Cape*	22.5	10.6	48	-2.1
*Free State*	51.1	7.2	56	-1.8
*Western Cape*	35.9	10.7	66	2.7
*Optimum climate requirement*	100.0	22.0	85	13.4

BPD = Black pod disease

#### 3.5.5 What level of black pod disease outbreak should farmers and investors expect in KwaZulu-Natal?

**Fig 9:**
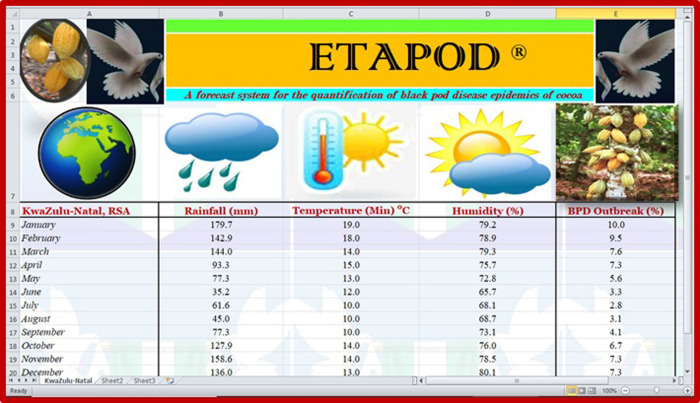
The pre-estimated trend of black pod disease outbreak in KwaZulu-Natal.

The months of January to May and September to December would be more challenging for intending cocoa farmers and investors in KwaZulu-Natal, as black pod disease activities would be high within these periods (Table 11 and Fig 9). The disease was estimated to be at its peak in the month of January (10.0%) and at its lowest in the month of July (2.8%), as shown in Table 11 and Fig 9 below.

**Table 11:** Pre-estimation of black pod disease burden in KwaZulu-Natal.

KwaZulu-Natal	Rainfall (mm)	Temperature (Min)°C	Humidity (%)	BPD Outbreak (%)
*January*	179.7	19.0	79.2	10.0
*February*	142.9	18.0	78.9	9.5
*March*	144.0	14.0	79.3	7.6
*April*	93.3	15.0	75.7	7.3
*May*	77.3	13.0	72.8	5.6
*June*	35.2	12.0	65.7	3.3
*July*	61.6	10.0	68.1	2.8
*August*	45.0	10.0	68.7	3.1
*September*	77.3	10.0	73.1	4.1
*October*	127.9	14.0	76.0	6.7
*November*	158.6	14.0	78.5	7.3
*December*	136.0	13.0	80.1	7.3

BPD = Black pod disease

### 3.6 Places to explore in KwaZulu-Natal for cocoa farming

The places to explore for cocoa farming in KwaZulu-Natal province of the Republic of South Africa were listed with their geographical coordinates in Table 12 below. Some of the fertile areas for possible establishment of cocoa farms include: Amajuba, Ballito, Berea, Durban, eSikhawini, Glencoe, etc. considerations for the establishment of cocoa farms within any of these areas should include the availability nearby river source to boost farm irrigation during the drier months or presence of high level of monthly rainfall or precipitation. In any case, the mean rainfall value for KwaZulu-Natal is sufficient enough to sustain commercial cocoa farming, even though plans for setting up water irrigation were not included in the initial budget for setting up of the cocoa farm.

**Table 12:** Sites to explore for cocoa farming in KwaZulu-Natal, Republic of South Africa.

**No**	**Cities**	**Latitude**	**Longitude**
1	Amajuba	27.7356°S	30.1354°E
2	Ballito	29.5390°S	31.2144°E
3	Berea	29.8519°S	30.9934°E
4	Dundee	28.1668°S	30.2337°E
5	Durban	29.8579°S	31.0292°E
6	Ekuvukeni	28.4675°S	30.1551°E
7	eMkhomazi	30.2067°S	30.7978°E
8	Empangeni	28.7620°S	31.8933°E
9	Eshowe	28.8865°S	31.4699°E
10	eSikhawini	28.8710°S	31.8996°E
11	eThekwini	29.8667°S	31.0167°E
12	Glencoe	28.1783°S	30.1470°E
13	Greytown	29.0642°S	30.5928°E
14	Hluhluwe	28.0190°S	32.2676°E
15	Howick	29.4780°S	30.2306°E
16	iLembe	29.2733°S	31.1425°E
17	Kokstad	30.5472°S	29.4241°E
18	KwaDukuza	29.3282°S	31.2895°E
19	Margate	30.8636°S	30.3705°E
**No**	**Cities**	**Latitude**	**Longitude**
20	Mondlo	27.9830°S	30.7177°E
21	Mooirivier	29.2082°S	29.9946°E
22	Mpophomeni	29.5682°S	30.1862°E
23	Mpumalanga	29.8129°S	30.6365°E
24	Mtubatuba	28.4179°S	32.1848°E
25	Ndwedwe	29.5169°S	30.9269°E
26	Newcastle	27.7580°S	29.9318°E
27	Pietermaritzburg	29.6168°S	30.3928°E
28	Port Shepstone	30.7414°S	30.4550°E
29	Richards Bay	28.7830°S	32.0377°E
30	Richmond	29.8720°S	30.2724°E
31	Scottburgh	30.2867°S	30.7532°E
32	Sisonke	30.1108°S	29.6601°E
33	Sundumbili	29.1337°S	31.3975°E
34	Ugu	30.5437°S	30.2748°E
35	Ulundi	28.3352°S	31.4162°E
36	uMgungundlovu	29.5093°S	30.1984°E
37	uMkhanyakude	27.6224°S	32.3295°E
38	uMzinyathi	28.5857°S	30.5588°E
39	uThukela	28.7192°S	29.6580°E
40	uThungulu	28.7005°S	31.5153°E
41	Utrecht	27.6586°S	30.3217°E
42	Vryheid	27.7695°S	30.7917°E
43	Zululand	27.8114°S	31.2943°E

## 4. Discussion

Some reports on the internet claimed that cocoa beans were currently produced in regions outside the natural ecological boundary or climate belt for cocoa production i.e., 20°N and 20°S of the equator, mostly within countries like Lesotho (29°S), Eswatini (26°S), Botswana (22°S), Namibia (22°S), and the Republic of South Africa (30°S), located in the fringes of Southern Africa (22° - 35°S of the equator). Judging by the country located farthest from the southern boundary of the climate belt for cocoa production, “*How possible is it for the clime of the Republic of South Africa to self-sustain commercial cocoa farming*?*”* “*Is it even possible for the cocoa tree to survive and produce quality cocoa beans outside its natural climate belt*?*”* The answer lies within the review of the climate of the Republic of South Africa (RSA), as the result suggest that cocoa could actually be cultivated beyond 30°S of the equator i.e., 10° farther from the southern fringe of its natural climate belt. The investigation also showed that the climate value for 5 months of the year i.e., January to March, November and December, was perfect and suitable for cocoa farming (i.e., 100% climate suitability rating), compared to that of the best cocoa producing nation of the world “Côte d’Ivoire” which could only boast of 6 months with 100% conformity to the benchmark climate requirements (i.e., June to November). Although, 7 out of the 12 months investigated had 43 to 71% climate suitability rating for cocoa production with an overall annual average rating of 71%, by logical reasoning, the current climate status of the Republic of South Africa would be appropriate and favorable for cocoa farming. The observations made were in accordance with the reports provided by the British media “British Pathé” [7], who claimed that cocoa was once cultivated in pre-colonial South Africa between 1920 and 1929, and it also validate, to some extent, the cocoa production statistics of the Republic of South Africa provided by the web media “Trading Economics” [6] in their 2022/2023 market analysis. The reason why cocoa farming was discontinued by farmers in RSA is still unclear, maybe their choice of cropping and crop production was influenced by financial costs for cocoa farm setup or heavy losses incurred by farmers after harvest as a result of massive annual crop loss due to disease infestation of cocoa pods/beans in the field/store houses or agro-ecological and climate-related constraints or even market factors which could simply be a case of demand and supply. In any case, the agro-ecological system of RSA has been analyzed and adjudged suitable for cocoa production; therefore, investors and farmers can invest in cocoa farming with ease provided they follow all the recommendations and highlights provided in this research.

So, *“Is the clime of the Southern parts of the African continent conducive for cocoa farming*?*”* A close comparison of the mean annual climate values of the Republic of South Africa (located at the fringes or extreme boundary of Southern Africa) and that of the benchmark climatic factors will suffice as a yardstick to draw any logical conclusion to the question raised. The climatological survey showed that the annual minimum temperature (10.8°C), mean temperature (18.3°C), and maximum temperature (25.8°C) of the Republic of Africa (RSA) were within the range of the standard climate requirements for cocoa farming i.e., 10°C (Benchmark minimum temperature), 18°C (Benchmark mean annual temperature) and 35°C (Highest tolerable temperature limit for cocoa production). Also, the air-water vapour saturation limit (62%) and monthly sunshine duration (8.4 hours per month) were also favorable for cocoa farming, as the estimated values were well within the standard climate requirements for cocoa production, but there was a shortfall in annual precipitation (463.6 mm) and monthly rainfall distribution (almost 5days per month). A functional alternative for water supply to farms within Southern Africa is currently in place to offset the deficiency in rainfall i.e., farm irrigation systems are currently operated by farmers to boost crop productivity as ordered by “The Water Act of the Republic of South Africa, No. 54, promulgated into law in 1956 [29]. Therefore, the data generated from the climatologic study conducted was substantial and convincing enough to support the logical conclusion that the climate and vast lands of the Southern fringes of the African continent was conducive enough for cocoa farming. This inference was also supported by the recent boost in the cocoa production capacity of Madagascar and Angola, ranked 8^th^ and 15^th^ in Africa and 20^th^ and 39^th^ in the world, respectively [10]. Also, the ancient claims of some British explorers establishing cocoa plantations within the pre-colonial South African country [7], also corroborates this logical conclusion. *“Where can commercial cocoa farms be established in the Republic of South Africa*?*”* Almost all the agricultural areas in the Republic of South Africa were able to support and sustain cocoa cultivation to some extent, but the province with the best clime for maximum cocoa production was KwaZulu-Natal, as it was 100% suitable for the establishment of new cocoa farms. Although, arable and fertile agricultural lands in other provinces like Gauteng, Mpumalanga, Eastern Cape, Limpopo, and Northwest can also be considered by investors who are willing to maximally explore the terrain of the Republic of South Africa for cocoa production, no assurance is given for optimum cocoa production output within these areas. This finding was in line with the reports made by Adey *et al*. [30], Trade and Investment KZN [31], and KZN Top Business [32], who stated that KwaZulu-Natal has long been recognized as the “food basket” of the Republic of South Africa, particularly with regard to vegetable production and dairy farming, making it the most productive region for agricultural activities in the Republic of South Africa. The Food and Agricultural Organization of the United Nations (FAO) has also joined the race in the establishment of projects in the Republic of South Africa that would increase food production and further strengthen food security within the country. One of the most remarkable achievements of FAO was the initiation of the “inclusive and sustainable food systems” in rebuilding the economies of KwaZulu-Natal (KZN) and Guateng provinces, with the establishment of projects like TCP/SAF/3805/C4, TCPF, TCP/SAF/3002 etc. [33]. Also, the idea of a proposition for cocoa farm establishment in other zones apart from KwaZulu-Natal was supported by the agricultural analysis of major provinces within the Republic of South Africa provided by Agribook [34].

Furthermore, answers were provided to some of the pertinent questions raised in the introductory aspect of this article (Section 1). The most pertinent question raised was: *“What could be the reason behind the global shift in the natural climate belt for cocoa production*?*”* The only logical rationale behind the recent extension of the natural climate belt for global cocoa farming, down to 30°S of the equator, could be as a result of the recent climate change around the world as it was proven that the climate of the Republic of South Africa was affected to some extent by global warming too. *“Is it possible that theories or facts about the geographical distribution of climate-restricted species like cocoa could be wrong*?*”* It is expected that many ecological barriers would be broken for some climate sensitive plants, animals, micro-organisms and other macro-organisms too. Therefore, scientists around the world should brace up for the unexpected because a possible alteration of the ecological barrier for global species distribution could be beneficial and at the same time harmful to life. *“Has the recent scientific advancement in genetic engineering conferred ubiquity to cocoa hybrids used as seed plants*?*”* There have been no documented reports to support or disprove this claim. *“Is the cocoa species dynamics altered by the recent climate change*, *such that it is no longer threatened by climate restriction*?*”* To some extent “YES”, the current change in climate distribution around the world would cause a slow or gradual redistribution of all living species such that some species can even thrive in areas where they were never reported to be seen before. *“Are theories of ecological distribution of climate-sensitive species becoming outdated*? “No”, they only need to be re-modified to suit the current climate classification. *“What if the current global climate change was indeed re-organizing global species distribution such that ecological barriers are broken and an unusual facet for new species evolution theory discovered*, *based on climate re-alignment with the past*?*”* This is indeed very possible, but the fact still remains that the recent global climate change could re-direct the focus of archaeologists around the world to unearth facts that could reshape species evolution and the whole of science forever. Nonetheless, it is noteworthy to state that the period for major cocoa production (i.e., major harvesting period) in Africa is between March and October (corresponding to the period for rainy season in Africa), where most indigenous cocoa farmers harvest more than 75% of their cocoa pods from the field, while the minor harvesting period starts from November and ends in April, during which less than 25% of the total crop volume is harvested. The period for minor harvesting of cocoa pods from the field span across the dry season through to the earliest period of the wet season. In any case, the optimum period for bountiful cocoa production across the African continent is between July and August [20].

The epidemiological studies conducted was able to validate the results of the climatological analysis carried out earlier and also provide answers to some of the questions raised in this report. For example: *“How accurate and reliable is the climate suitability analysis carried out on the Republic of South Africa*?*” “Is it even possible for the pathogens of cocoa to exist outside an environment that is unfavorable to their host plant(s)*?*”* The study conducted showed that none of the top 20 cocoa producing nations of the world had 0% prediction for black pod disease (BPD) occurrence. In Africa, indigenous cocoa farmers in countries like Ghana, Côte d’Ivoire, Nigeria, and Cameroon are seriously threatened by annual black pod disease outbreak, as the disease is capable of causing 100% loss of farm produce. Therefore, the presence of cocoa pestilence within these countries is clear indication that the climate suitability studies carried out on the Republic of South Africa was indeed accurate and reliable. This report was supported by the research conducted by Etaware [20, 35], and Etaware and Adedeji [21] in Nigeria, and that of Akrofi [36] and Opoku *et al*. [37] in Ghana. The research findings was also corroborated by the current reports of AGRIGOLD [38], who stated black pod disease outbreak in most cocoa plantations in Ghana was the major reason why Ghana’s cocoa contribution to the global market dropped, beginning from 1985 when it overtook the cocoa swollen shoot disease (CSSD) as the most devastating disease of cocoa in Ghana. Therefore, *“If truly cocoa can be cultivated in the Republic of South Africa*, *would it be possible that the country will share similar disease epidemiology with other cocoa producing nations within and outside Africa*?*”* The results obtained from the epidemiological studies carried out within the clime of the Republic of South Africa showed that black pod disease outbreak was expected to occur between January-April and October-December. The disease was estimated to be more intense in cocoa plantations established in KwaZulu-Natal (8.6%) and Eastern Cape (6.6%) provinces of the Republic of South Africa and lowest in Northern Cape Province (-2.1%). The high level of black pod disease outbreak estimated for KwaZulu-Natal Province is a confirmation that the region is indeed favorable and conducive for cocoa farming just like Ghana, Côte d’Ivoire and Nigeria too.

## 5. Conclusion

The climate analysis and disease epidemiological studies carried out in this research was a pointer to the fact that farmers and investors looking for arable land to establish new cocoa farms should consider investing their finance in the Republic of South Africa (RSA), most especially KwaZulu-Natal Province, and its neighboring countries, as these lands might be underutilized and could be even more fertile, productive and profitable for cocoa farming. Based on the climatological and epidemiological analysis carried out in this research, it is safe to say that the agro-ecological system of RSA tend to become more favorable for cocoa production as the years goes by, based on the observations and inference drawn from the 1^st^ and 3^rd^ decile analysis of climate data of the Republic of South Africa provided earlier in this research.

### 5.1 Recommendations

In the Republic of South Africa, agriculture is generally threatened by poor soil structure and drought. Cocoa farming is fast been neglected not only in the country but also other parts of Africa where the crop thrives better like Nigeria, and Ghana [10], which were once leading cocoa producing countries in the world. South Africa has a semi-arid land with almost 65% of its land mass threatened by drought and poor soil structure. The implementation of a new low-cost strategy for soil improvement alongside the already structured irrigation channels will help increase the chances of the country to become a major contender, not just as cocoa producers, but in all spheres of agriculture. Rejuvenation of cocoa farming in the Republic of South Africa will help boost the economy’s financial strength and also help strengthen the value of their currency against other foreign currencies due to massive export of agricultural produce. The use of soil additives and gene engineering to combat all manner of plant diseases is a new theme that is still gaining more grounds and attention in the world of science and agriculture [39]. Furthermore, these bio-additives can help increase the water holding capacity of the already deformed South African soil. It can also reduce soil plasticity, increase soil aeration and beneficial microbial colonies, and also help maintain soil stability for long term bountiful produce of cocoa. The process is not only cost effective, but it will reduce the level for forest encroachment in the Republic of South Africa.
